# Water-soluble contrast agent use in adhesional small bowel obstruction: a survey of surgical practices and clinical trial considerations

**DOI:** 10.1308/rcsann.2024.0121

**Published:** 2025-06-11

**Authors:** K Aimar, J Walshaw, A Peckham-Cooper, N Smart, L Magill, MJ Lee

**Affiliations:** ^1^ Department of General Surgery, University Hospitals Birmingham NHS Foundation Trust, UK; ^2^ Leeds Institute of Medical Research, University of Leeds, UK; ^3^ Department of Emergency General Surgery, Leeds Teaching Hospitals NHS Trust, UK; ^4^ Department of Colorectal Surgery, Royal Devon and Exeter NHS Foundation Trust, UK; ^5^ Birmingham Clinical Trials Unit, University of Birmingham, UK; ^6^ Department of Applied Health Sciences, University of Birmingham, UK; ^7^ Department of Trauma and Emergency Surgery, University Hospitals Birmingham NHS Foundation Trust, UK

**Keywords:** Small bowel obstruction, Contrast, Gastrografin, Survey, Feasibility work

## Abstract

**Background:**

The role of water-soluble contrast agent (WSCA) as a therapeutic tool in the nonoperative management of adhesional small bowel obstruction (ASBO) remains indeterminate. This survey aimed to understand current surgical practices in WSCA use in the conservative approach to ASBO, and to evaluate key design and feasibility factors to inform a future controlled trial of WSCA in ASBO.

**Methods:**

This study is reported in line with CROSS guidance. The survey consisted of 16 questions organised into three sections: respondent characteristics, current decision-making and WSCA use, and opinions on future trial. Pilot testing was conducted before online distribution to surgeons in the UK delivering Emergency General Surgery. Descriptive analysis was performed.

**Results:**

Of 73 total respondents, 52 (71.2%) were consultants. When treating ASBO conservatively, 80.8% (59/73) reported using WSCA in more than half of cases, but there was wide variation in timings, use and repeat challenges. Between 71.2% and 89.0% of respondents affirmed their willingness to adhere to specific trial protocols. Regarding feasibility, 76.7% (56/73) of respondents agreed or strongly agreed that they would be willing to recruit to a trial and 75.3% (55/73) agreed or strongly agreed that their unit would be able to deliver a trial.

**Conclusions:**

The survey revealed widespread acceptance of WSCA as a therapeutic tool in ASBO among emergency general surgeons. Although practices around its administration varied, there was a clear readiness to adopt standardised protocols. The majority of respondents expressed support for a controlled trial comparing WSCA against a placebo.

## Introduction

Adhesional small bowel obstruction (ASBO) imposes a considerable burden on patients and healthcare systems.^1,2^ Intestinal blockage accounts for 60% of emergency laparotomies performed in the UK.^3^ Adhesions represent the most common aetiology, responsible for approximately 55% of all small bowel obstructions.^4,5^ The Bologna guidelines, developed by the World Society of Emergency Surgery’s ASBO working group, detail two primary management strategies: operative intervention and conservative (nonoperative) treatment.^6^ Emergency surgery is essential for patients exhibiting features of peritonitis, bowel strangulation or ischaemia. Nonoperative management is preferred for cases without these complications and key elements of this approach include fasting, nasogastric tube insertion and fluid and electrolyte correction.^7^

An additional element of the conservative approach is the use of a water-soluble contrast agent (WSCA). The potential role of WSCA in ASBO has garnered significant attention, resulting in multiple meta-analyses evaluating its diagnostic and therapeutic applications.^8–12^ WSCA’s diagnostic role has been established, with its reliability in predicting the need for surgery acknowledged and incorporated into the Bologna guidelines’ diagnostic algorithm.^6,8^ However, the therapeutic advantages of WSCA for treating ASBO remain uncertain. Randomised controlled trials have yielded mixed results; although WSCA has been shown to reduce hospital length of stay and time to symptom resolution in numerous studies, such trials remain divided on whether it significantly reduces the need for surgery.^13–19^ Other studies have found no therapeutic advantage of using contrast media in ASBO.^20,21^ The research conducted so far has been limited in scale and varied in methodology. There is a need for a larger, more rigorous trial, but it is essential to first understand the current applications and perceptions of WSCA in clinical practice.

This survey aims to evaluate current practices in ASBO management and WSCA use among surgeons, and to assess key factors influencing the design and feasibility of a future controlled trial comparing WSCA with a placebo in the treatment of ASBO.

## Methods

### Study design

This study is reported in line with the Consensus-Based Checklist for Reporting of Survey Studies (CROSS) checklist.^22^ Surgeons in the UK who are practising at the level of Core Surgical Trainee (CST) or higher, delivering Emergency General Surgical services, comprised the target population for this cross-sectional study. The questionnaire, created using Microsoft Forms, was divided into three sections and contained a total of 16 questions (Appendix). The first section included two questions about respondents’ characteristics, specifically their training level and primary area of specialisation. The second section consisted of six questions designed to assess current decision-making practices for treating ASBO, as well as the use of WSCAs. The third section featured eight questions dedicated to exploring surgeons’ views on trial design, with a focus on protocol details and feasibility. In this section, respondents indicated their level of agreement with each statement on a five-point Likert scale ranging from ‘strongly disagree’, ‘disagree’, ‘neutral’, ‘agree’ or ‘strongly agree’.

The survey included a plan for a proposed double-blind, placebo-controlled, randomised trial (Table 1). Eligible participants described would be adults with computed tomography (CT)-proven ASBO, no features of ischaemia and the ability to undergo surgery if necessary. Patients with a hostile abdomen would be excluded. Participants would undergo a period of conservative management and be randomised to receive either WSCA or a placebo. The treating team would monitor progress and the decision to operate would be made by the surgeon based on specific triggers: failure to resolve by day three or the development of signs suggesting ischaemia.

**Table 1:** Outline of a proposed trial of WSCA in ASBO

Proposed trial:	Double-blind, placebo-controlled, randomised trial
Inclusion criteria:	Adults with CT-proven ASBO, no features of ischaemia on CT and able to undergo surgery if necessary (indicated by clinical frailty score ≤5 or ASA grade III or better)
Exclusion criteria:	Patients with a hostile abdomen
Procedure	
1) Recruitment after CT confirmation of diagnosis. 2) Double-blinded randomisation to either the treatment or placebo arm. 3) After a period of conservative management (intravenous fluids, analgesia and nasogastric tube insertion), the allocated therapy is administered (either 100ml oral gastrografin or 100ml of thickened, aniseed-flavoured water), and the nasogastric tube is spigotted to allow the fluid to travel distally from the stomach. 4) Participants are monitored by the treating team. The decision to operate lies with the treating surgeon. Triggers for surgery: failure to resolve by day three post-diagnosis, or the development of features concerning for ischaemia.	

ASA = American Society of Anesthesiologists; ASBO = adhesional small bowel obstruction; CT = computed tomography; WSCA = water-soluble contrast agent.

### Survey development and pretesting

The questionnaire underwent a multistage development process, starting with the identification of broad concepts; then, through group consensus, specific questions were generated to evaluate each of the three sections of interest. Three researchers (MJL, APC, NJS) participated in this process. Before its launch, the questionnaire was pilot tested by five individuals.

### Survey administration

The survey remained open for a two-week period from 15–29 July 2024. It was distributed across social networks, in an Emergency General Surgery interest group, and through personal contacts.

### Data collection and analysis

Responses were captured automatically and exported to an electronic spreadsheet. Alongside the questionnaire answers, the survey start and finish times for each participant were logged. Respondents were required to submit a valid email address to confirm their eligibility in the target population and to identify any instances of duplicate submissions. Only completed questionnaires were included in the analysis. No link click tracking was used, so completion rate cannot be assessed accurately.

## Results

### Respondent characteristics

A total of 73 responses were submitted within the study period, with an average completion time of five minutes. Consultants comprised the majority of respondents (52/73, 71.2%) (Table 2). The level of clinical practice of other respondents included: Senior Registrar/Specialty Trainee (ST)7-8 (10/73, 13.7%), Registrar in training/ST3-6 (7/73, 9.6%), CST or equivalent (3/73, 4.1%), and Specialty and Associate Specialist (SAS) doctor (1/73, 1.4%). Respondents most commonly identified Emergency General Surgery (31/73, 42.5%), Colorectal Surgery (28/73, 38.4%) or Upper Gastrointestinal Surgery (12/73, 16.4%) as their main area of practice. Hepatobiliary Surgery was the primary area of practice for two respondents (2/73, 2.7%).

**Table 2:** Respondent characteristics.

Characteristic	Number of respondents (%)
Level of practice	
CST or equivalent	3 (4.1)
Registrar in Training/ST3-6	7 (9.6)
Senior Registrar/ST7-8	10 (13.7)
SAS	1 (1.4)
Consultant	52 (71.2)
Primary area of practice	
Emergency General Surgery	31 (42.5)
Colorectal Surgery	28 (38.4)
Upper Gastrointestinal Surgery	12 (16.4)
Hepatobiliary Surgery	2 (2.7)

CST = core surgical trainee; ST = specialty trainee; SAS = specialty and associate specialist doctor.

### Current decision making in ASBO

**Figure 1:**
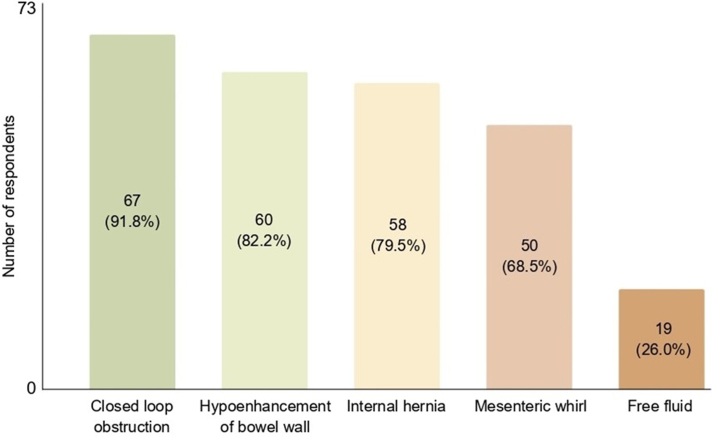
CT findings influencing decisions for surgery. CT = computed tomography.

Respondents were presented with a list of CT findings and asked to select all of the ones that would make them more likely to offer surgery rather than a trial of conservative management. The most common answer was closed loop obstruction, selected by 67/73 (91.8%) of respondents (Figure 1). This was followed closely by hypoenhancement of the bowel wall (60/73, 82.2%), internal hernia (58/73, 79.5%) and mesenteric whirl (50/73, 68.5%). Free fluid was selected less frequently (19/73, 26%).

Respondents had the option of entering findings that were not listed in the survey that might influence their decision to opt for surgery. Five respondents entered three additional CT findings: sharp transition point (3/5); acute massive enlargement of small bowel (1/5) and pneumoperitoneum (1/5). Other respondents submitted clinical factors that would raise the likelihood of them taking surgical approach: features of peritonism on physical examination (2/6); critical clinical condition as indicated by vital signs and blood test results (2/6); history of intra-abdominal adhesions (1/6) and no previous abdominal surgery, or so-called ‘virgin abdomen’ (1/6).

### WSCA use

**Figure 2:**
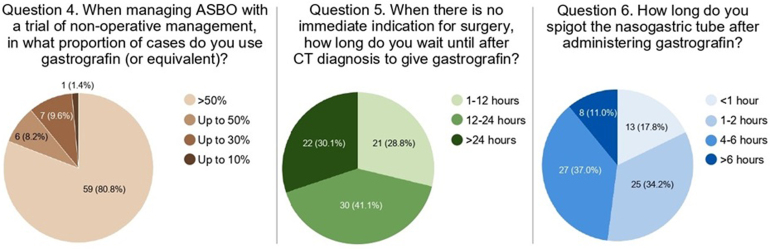
Current practices in WSCA use among survey respondents. ASBO = adhesional small bowel obstruction; CT = computed tomography; WSCA = water-soluble contrast agent.

The survey incorporated three questions to assess the use of WSCA in current clinical practice (Figure 2). When treating ASBO with a trial of nonoperative management, 59 respondents (80.8%) reported using gastrografin, or an equivalent WSCA, in more than half of such cases. The remaining respondents indicated less frequent WSCA usage, with six using it in up to 50% of cases, seven in up to 30% and one in up to 10%.

In the subsequent question, respondents indicated the timeframe within which they administer WSCA after radiographic diagnosis of ASBO suitable for a trial of conservative management. This interval was within 1–12 hours for 21 respondents (28.8%), within 12–24 hours for 30 respondents (41.1%) and over 24 hours for 22 respondents (30.1%). When asked how long respondents spigot the nasogastric tube for after administration of WSCA, most answers were divided between 4–6 hours (27/73, 37.0%) and 1–3 hours (25/73, 34.2%). Thirteen respondents (17.8%) spigot the tube for an hour, whereas eight (11.0%) spigot the tube for over six hours after WSCA administration.

Two questions in the survey evaluated respondents’ perceptions of WSCA use. In judgement of the statement ‘gastrografin challenge is useful as a predictor of surgery’, the proportion of those who agreed or strongly agreed (34/73, 46.6% and 28/73, 38.4% respectively) surpassed those who disagreed (3/73, 4.0%). Eight respondents (11.0%) were neutral and none strongly disagreed. When asked to assess the next statement, ‘gastrografin is useful as a therapeutic agent, independent of surgery’, 93.2% of respondents agreed or strongly agreed (33/73, 45.2% and 35/73, 48.0%, respectively). Four respondents were neutral (5.5%), one disagreed (1.4%) and none strongly disagreed.

### Opinions on future trial

**Figure 3:**
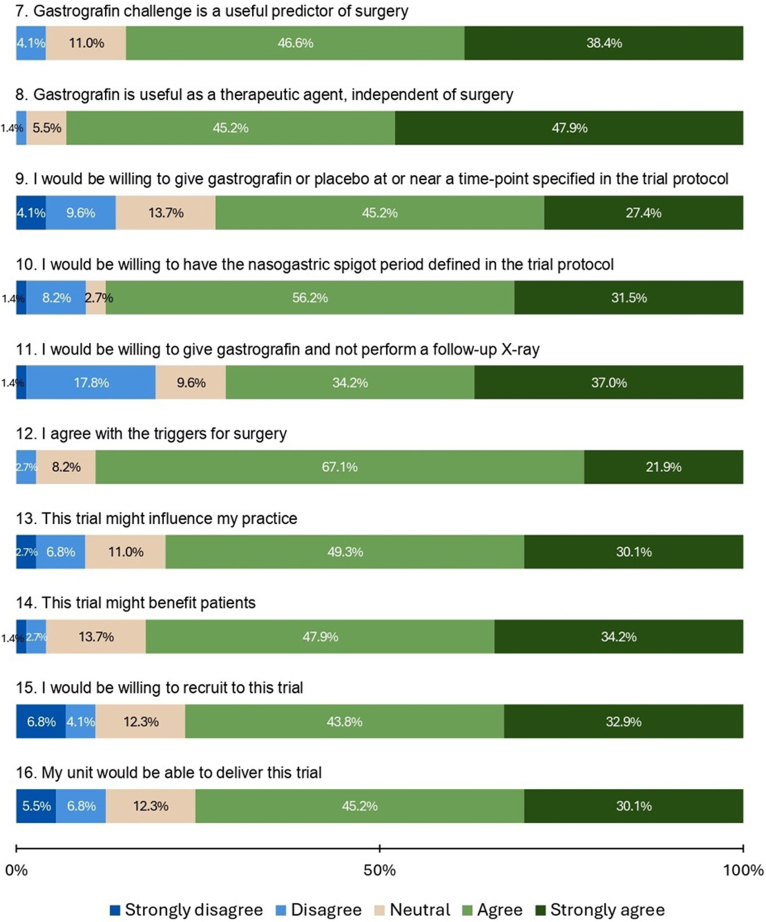
Respondent opinions on design, value, and feasibility of a proposed clinical trial of WSCA in ASBO. ASBO = adhesional small bowel obstruction; WSCA = water-soluble contrast agent.

In the third section of the survey, respondents were asked their opinions pertaining to the design, value and feasibility of a proposed clinical trial to evaluate WCSA use in nonoperatively managed ASBO. The proportion of positive (‘agree’ or ‘strongly agree’) responses exceeded the proportion of neutral and negative (‘disagree’ or ‘strongly disagree’) responses for each of the eight questions in this section (Figure 3). In regard to trial design, respondents indicated their willingness to adhere to the trial protocol across four areas: timing of gastrografin/placebo administration (27.4% strongly agree, 45.2% agree, 13.7% neutral, 9.6% disagree, 4.1% strongly disagree), timing of nasogastric tube spigotting (31.5% strongly agree, 56.2% agree, 2.8% neutral, 8.2% disagree, 1.4% strongly disagree), foregoing follow-up x-ray (37.0% strongly agree, 34.2% agree, 9.6% neutral, 17.8% disagree, 1.4% strongly disagree) and specific triggers for surgery (21.9% strongly agree, 67.1% agree, 8.2% neutral, 2.7% disagree, 0% strongly disagree).

Eighty-two percent of respondents agreed or strongly agreed that the trial may benefit patients. The final survey questions, pertaining to feasibility, asked respondents whether they would be willing to recruit to the trial – 76.7% (56/73) agreed or strongly agreed, 11.0% (8/73) disagreed or strongly disagreed – and whether their unit would be able to deliver the trial – 75.3% (55/73) agreed or strongly agreed, 12.3% (9/73) disagreed or strongly disagreed.

Respondents were given the opportunity to provide comments or feedback on the proposed trial. Three respondents recommended that the term ‘hostile abdomen’ should be defined in the exclusion criteria. Four respondents indicated that WSCA is considered the standard of care, or is part of existing local protocols, which could complicate placebo administration and lead to concerns about the implications of withholding WSCA. Two respondents expressed that waiting three days to proceed with surgery after failed conservative management is too long.

## Discussion

Our survey revealed a high level of acceptance of WSCA as both a predictive and therapeutic tool in routine clinical practice. In addition, the overwhelming majority of respondents expressed support for a future controlled trial comparing WSCA with a placebo, indicating readiness to adhere to trial protocols and a belief in the potential patient benefits. Current clinical practices regarding WSCA usage were heterogeneous, particularly regarding the timing of contrast administration after ASBO diagnosis and the timing of nasogastric tube spigotting.

The questionnaire yielded valuable insights into the use of WSCA in current surgical practice, enabling comparisons with earlier survey research and actual usage statistics. The strong consensus among respondents on the diagnostic and therapeutic value of WSCA in ASBO supports the finding that 81% use WSCA in the majority of such cases, with all respondents employing it at least occasionally. However, it is worth considering that support for, or self-reported use of, contrast media in ASBO may differ from its actual application in practice. A national audit revealed that while 97% of consultant surgeons would consider using WSCA in ASBO, a cohort study of the same target population found it was actually implemented in only 22% of cases.^4,23^ Additional research to identify and address the barriers to the adoption of WSCA in the management of ASBO may be warranted. Another area requiring further investigation is in the development of an evidence-based protocol for the administration of contrast media in patients with bowel obstruction.^24^ The variability in the timing of WSCA administration and nasogastric tube spigotting in our study highlights the absence of standardised practices. A clinical trial comparing gastrografin with placebo in patients with conservatively managed ASBO could provide evidence to support the development of guidelines for the effective use of WSCA.

The high level of agreement among respondents on the diagnostic utility of WSCA aligns with its established efficacy as a predictor of surgery, with studies indicating it has at least 92% sensitivity and 93% specificity for predicting the resolution of obstruction without surgical intervention.^8–10^ Interestingly, surgeons in this study showed even greater support for WSCA as an independent treatment modality despite research on its effectiveness in this role yielding inconsistent results.^13–16^ The strong endorsement of WSCA as a therapeutic tool by respondents may be a reflection of personal clinical experience rather than the current evidence base. Moreover, this level of acceptance raises the question of whether some surgeons may be reluctant to withhold WSCA in a placebo-controlled trial, and consequently make them less inclined to participate, as highlighted by some respondents in their survey feedback. A lack of individual equipoise may undermine recruitment efforts and influence surgeons’ decision of applying eligibility criteria.^25,26^

Overwhelmingly positive responses continued into section three of the survey, where most respondents indicated a willingness and ability to facilitate a clinical trial exploring WSCA use in patients with ASBO. The large proportion of respondents prepared to comply with defined trial protocols reflects a readiness to adopt standardised practices, which is essential both for the success of future trials and the implementation of clinical practice guidelines. Some respondents expressed concerns about a three-day trial of conservative management before converting to surgery, considering it too long. However, current evidence supports this as a safe interval, provided no other surgical indicators, such as ischaemia, emerge.^27–29^ The subset of respondents who were neutral or disagreed with the design, feasibility or value of further research in this area presents an opportunity to explore potential reservations, uncertainties and barriers.

The term ‘hostile abdomen’ as an inclusion or exclusion criterion for a trial would bring challenges. This may be hard to define until one has commenced the operation. Surgeons may have differing risk attitudes, and therefore have different thresholds for an anticipated ‘hostile abdomen’. This subjective approach to definition might lead to cherry picking of cases likely to have a favourable outcome, skewing the findings of a trial. An objective definition of likely hostile abdomen in ASBO will be required for future studies.

This survey provides useful insight into the use of a common intervention for ASBO, and is necessary to plan the development of future trials in the area. Surgical research has long been criticised. Emergency surgery is a relative research desert, and in particular need of high-quality trials. Surgeons have some particular challenges around equipoise, which have impacted the recruitment of randomised controlled trials.^25,26^ Appreciating where surgeon equipoise and acceptance lie is essential to planning a deliverable trial.^30^ Additionally, by capturing expert perspectives on clinically meaningful effects, surveys can help to determine the sample size needed to detect those important factors.

An accurate interpretation of the study’s findings involves acknowledging both its strengths and limitations. The study’s focus on surgeons with active roles in Emergency General Surgery ensured that participants were qualified to contribute informed insights on WSCA use in ASBO treatment. The predominance of consultants among respondents suggests that the findings reflect the practices and views of expert decision makers. Moreover, distributing the survey across multiple internet-based platforms likely broadened its reach and respondent diversity, leading to a more representative sample of the target population. However, this approach presents two potential limitations. First, selection bias may have influenced the findings, as those active in relevant interest groups or within the researchers’ networks may be more inclined to favour WSCA use in ASBO or support a trial on the topic. The absence of a measured response rate further complicates this issue. Second, the electronic distribution of the questionnaire via a link could allow multiple submissions from the same respondent or participation by ineligible individuals, though the inclusion of email address collection helped mitigate this risk. The modest sample size is also acknowledged as this may limit the representativeness of the results. One strength of the study was that all 73 surveys were fully completed, ensuring comprehensive data from each participant. Although view and completion rates were not measured, the survey’s brief format, requiring an average of five minutes to complete and containing no mandatory free-text answers, likely reduced the barriers to questionnaire completion.

In conclusion, this study reveals that UK surgeons experienced in Emergency General Surgery strongly endorse and frequently use WSCA for nonoperative management of ASBO. Although there is variability in current WSCA administration practices, there is a notable readiness to implement standardised procedures. Furthermore, there is considerable enthusiasm for a future controlled trial evaluating WSCA against placebo in ASBO treatment, with participants expressing belief in the feasibility of such research and its potential benefits for patients.
